# Medical Opinion and Movement

**Published:** 1908-08-22

**Authors:** 


### MEDICAL OPINION AND MOVEMENT.

THE advantages and disadvantages of spinal anæsthesia produced by stovaine are fully discussed in the *Medical Magazine* by Mr. McGavin. After a description of the technique of the induction, he urges several points of superiority of this method over the use of general anæsthetics. The terror of loss of consciousness he holds to be a material factor in promoting shock in some patients, and especially in women. This fear is soon dispelled when anæsthesia supervenes, and the patients find themselves able to converse with their nurses or medical men. Pre-operative and post-operative starvation are unnecessary, and pulmonary complications are equally avoided; in both cases it will be conceded that a good deal is thus gained. Then again if permission should be required for some extension or alteration of the proposed operation due to disclosures during its progress, consent can be asked of the patient there and then for the suggested new course. Mr. McGavin doubts the common belief that shock is diminished by profound general anæsthesia, but he holds strongly that it is so during spinal analgesia. He urges that in military surgery, in serious railway wrecks, and similar emergencies, many lives might be saved by thus anæsthetising patients before removing them to hospitals. He is also enthusiastic as to the very complete muscular relaxation which is obtained, which greatly facilitates laparotomies, radical cure of hernia, and all rectal operations especially. The avoidance of post-operative vomiting is another great gain from the points of view of both surgeon and patient, and as no nurse is required to sit by the latter's bed on his return to the ward, advantage accrues also in that way.

THE disadvantages include occasional air hunger for a short time (without cyanosis), and in some cases a second injection necessary before the completion of the operation. Pyrexia to 100° or 101° F. is seen in most cases for the first forty-eight hours. Headache and backache are not uncommon, but yield to aspirin. Nausea, sickness, rigors are all rare. One drawback that has been noticed is the flabby appearance of hæmorrhoids, which are not thus clearly marked off from surrounding mucous membrane as when congested by etherisation. The Trendelenburg position is contraindicated, lest the stovaine gravitate towards the brain, and the lithotomy position is not likely to be regarded with favour by a conscious patient. Failure to enter the theca or to maintain rigid antisepsis are, of course, not to be blamed on to the method, and are in Mr. McGavin's opinion both avoidable by reasonable skill. The presence of a bedsore is naturally an objection, but even that difficulty could be got over, he thinks, by cauterising it. Whether or not spinal anæsthesia will prove of value in midwifery it seems not yet possible to say. One observer thinks it increases uterine contraction, and may therefore be contraindicated in version and other obstetric operations. Dangerous extension of the analgesia upwards is probably due to overdosage or carelessness. Whether the spinal method is or is not more dangerous than general anæsthesia, especially by chloroform, is regarded as not yet proved. Meanwhile patients themselves are almost without exception, it is alleged, enthusiastic as to its merits.

FROM the clinical point of view gonorrhœal arthritis can be recognised under four different forms, namely, hydrarthrosis, sero-fibrinous arthritis, empyema, and phlegmonous arthritis. Preiser (*Munch. Med. Woch.*) gives the result of his observations on 83 severe cases of the last group. He believes that unless intervention be prompt in these cases the prognosis is extremely grave. There is but slight articular effusion, swelling for the most part being peri-articular: the skin is red and œdematous and there is much pain in the joint. Such conditions can be readily distinguished from ordinary rheumatism, for fever is slight or even absent, the affection is mon-articular, and treatment by salicylates has no effect. The treatment he advises consists in first overcoming the inflammatory condition, and next in preventing deformities of the joint. Hot baths should be given, even in the acute stage, and these should be followed early by massage and movement. Later, to prevent deformities, a plaster splint should be ordered. In no case has this treatment been followed by any aggravation of the symptoms or by the production of swellings in other joints. Treatment by keeping the affected part at rest proved useless.

H. EYSSEL, in the *Munch. Med. Woch.*, mentions a method of treating certain forms of syphilitic lesions which he has found extremely useful. Originally he used the treatment for children, the subjects of hereditary syphilis and suffering from "snuffles." Three times a day one and a half grains of calomel and sugar of milk in equal parts are passed into each nostril by means of an insufflator. A rapid improvement in the patient's condition follows the continuance of this treatment. The author has more recently applied the same treatment to adults suffering from mucous plaques and ulcerations of the nasal mucous membrane, tonsils, and soft palate. If the patient takes a deep breath the powder easily reaches the larynx and trachea. In the case of adults the dose of the powder is correspondingly increased in accordance with their age and toleration of the drug. There are said to be no troubles arising from this form of treatment and no local irritation unless iodides be administered at the same time. Stomatitis occurs but rarely if suitable care be taken of the mouth. The author claims that syphilitic manifestations in other parts of the body always disappear after this method of treatment has been employed.

THE "summer diarrhœa" of children is such a scourge in all countries, that any form of treatment which holds out hope of reducing the death-rate from this disease is worthy of a trial.

Houssaye in the *Gazette des Hôpitaux* calls attention to a form of treatment which he claims to have used with the greatest success. His method consists in washing out the lower bowel with ordinary claret. The wine used is of the vintage of the current year, old wine being too strong in alcohol. The wine is given warm, in a steady stream, through a two-way cannula, not as an injection. Two or three "lavages" are given each day, consisting on the first day of two pints each, and four pints each on successive days. This is continued until the stools once more become normal. The treatment is equally efficacious in older children, but should then not be entrusted to the nurse, as in such cases it is more difficult to carry out the "lavage," since the patients are more refractory. Houssaye claims that this treatment is simple, inexpensive, and efficacious. As far as this country is concerned, the plea of inexpensivenesss will not hold good; and, moreover, what passes here for *vin ordinaire* is in many cases not the product of the grape at all, but of the chemical laboratory. It seems, therefore, that to carry out this method of treatment it would be safer to use a solution of alcohol and tannin of known strength when the wine obtainable is not above suspicion.

---

Every new fact discovered in regard to variations in the constituents of the blood in health and disease adds an important brick to the structure of medical science. Liepmann and Stern have shown that the glucose content of the blood in normal individuals is remarkably constant, and varies between the extremes of .065 per cent. and .105 per cent. On the other hand Lüthje finds that with the dog the glucose content is almost the same as in man, but that it varies according to the external temperature. Cold increases the glycæmia, heat diminishes it. But Liepmann and Stern have further demonstrated that during the fever of pneumonia in man there is a hyperglycæmia. Following up these researches, Hollinger has examined the glycæmic condition in a large number of patients suffering from febrile affections of all kinds; pneumonia, scarlatina, enteric, chronic suppuration, etc. He finds that in all cases the fever is accompanied by a more or less intense hyperglycæmia, amounting sometimes to .16 per cent. Seeing that diabetics often show a diminished glycosuria during a febrile attack, the exact opposite might have been expected. It appears, however, that just as the dog requires more glucose to burn, in order to compensate increased radiation in the cold, so the febrile patient, producing an excess of heat to maintain the abnormal temperature, burns more glucose also, to compensate in the same way the excessive radiation.

---

The colloid preparations of the metals have recently received a good deal of attention at the hands of French physicians as therapeutic agents, and in particular the colloid form of silver in solution. Professor Jeannin gives an enthusiastic account of the efficacy of this preparation, so-called collargol, in puerperal fever. Collargol can be prepared by chemical methods, but the purest and most active product is obtained electrically. It is not only a powerful bactericide, but it is also supposed to possess catalytic diastatic properties, by which it acts as a stimulant to the physiological reactions of the organism. It can be administered by intra-muscular or intravenous injections. Professor Jeannin decides in favour of the latter method on account of its greater rapidity and intensity of action. He uses a 1 per cent. solution, and injects 10 to 15 cubic centimetres at a time. The injections should be repeated every second or third day according to the course of the disease. The injection should be carried out by an oblique puncture directly through the skin into one of the veins at the bend of the elbow. The injection is usually followed by an immediate momentary rise of temperature, accompanied by a rigor more or less intense. In favourable cases this is followed by a gradual fall of the temperature with marked amelioration in the general condition of the patient. In the maternity hospital of Lariboisière he has used the treatment in forty-three cases of severe puerperal fever, and of these thirty-three recovered and ten succumbed, giving a proportion of recoveries of seventy-six per cent. He advocates the treatment in all puerperal infections, whenever the infection is of a serious septicæmic type, or when the infection appearing at first to be localised tends to become generalised. He does not claim it to be an absolute panacea for puerperal fever to the exclusion of all other methods of treatment, but is convinced that it is a powerful weapon in conjunction with other therapeutic means.

---

Dr. W. Gordon, of Exeter, has drawn attention to an interesting clinical phenomenon which he has observed in a large number of cases of cancer, and which may prove of some utility in the diagnosis of the disease—namely, a diminution in the cardiac dulness. The number of cases upon which he makes his report amounts to 103. They were all such as to raise, in a greater or less degree, the suspicion of cancer, and in all of them the reduction in the area of dulness could not be fairly accounted for by ordinary causes. With the patient in the recumbent position Dr. Gordon takes as the area of normal cardiac dulness the space limited above by the third costal cartilage, to the right by the midsternum, and measuring about 3 inches across at the level of the fifth costal cartilage. The diminished dulness such as he finds in these cases begins above at the fourth costal cartilage, has a right limit to the left of the sternal border, and measures less than 2 inches, and in many cases less than 1 inch across at the level of the fifth costal cartilage. There are often associated a small feeble low tension pulse and weak heart sounds. Of the cases which were proved either during life or post-mortem to be cancerous, about 90 per cent. showed diminished cardiac dulness. On the other hand, of cases which were doubtfully cancerous, and which subsequent history proved not to be so, only 24 per cent. showed diminished cardiac dulness. Dr. Gordon admits that in the majority of cases at the time of observation the disease was already so advanced that operation was no longer possible, but he finds that diminished dulness commonly precedes emaciation and cachexia. He suggests that this clinical sign may be due to a flaccidity of the heart muscle or to the heart being less well filled than in health, or the lungs may lose some of their elasticity and so cause a spurious emphysema. Lastly, it has been shown in advanced cases of cancer that the heart does actually become reduced in size.

---

The researches of the Royal Commission on Tuberculosis have clearly established the possibility of human infection with tuberculosis from tuberculous cattle, either by the milk or by the ingestion of infected meat, and the experimental researches of Calmette and others on animals have shown that the ingestion of tubercle bacilli may lead to an infection of the tracheo-bronchial glands and of the respiratory system without giving rise to any tuberculous infection of the intestinal tract. These facts have induced the view that infection *viâ* the alimentary canal plays a more important *rôle* in the spread of tuberculosis than direct inhalation of the bacilli from the air. In a paper read before a recent meeting of the Societé Médicale des Hôpitaux, M. G. Küss combats this view. By experiment on guinea pigs he finds that, contrary to the experiences of Peterson, Cadéac, and Calmette, it is quite easy to infect these animals with tuberculosis by making them inhale the dried powder of tuberculous sputum, and that it is possible in this way to reproduce the most common anatomical forms of primary infantile tuberculosis. Provided the inhalations are not too intense, they produce a ganglio-pulmonary infection characterised by subpleuritic caseous nodules, accompanied by extensive fibro-caseous changes in the tracheo-bronchial glands, and he maintains that these experimental lesions are in all respects analogous to those commonly found in children. M. Comby expressed himself in complete accord with the views of M. Küss, and considered that clinically and pathologically the evidence was in favour of infection by inhalation rather than by ingestion. In 1,432 autopsies on infants, he has found pulmonary tuberculosis in 523, and in not one of these cases has he discovered a primary tuberculosis of the intestine. He considers, therefore, that the danger of infection does not lie in the milk but in the air, and in infected surroundings. M. Comby maintains that he has always found tuberculosis among the relatives of tuberculous children, and thinks that prophylaxis lies in preventive methods against human contagion. Such a view must be held at the present time to be "reactionary" in extreme, but the problem of infection is undoubtedly still *sub judice*, and meanwhile the only practical course of prophylaxis is to keep guard upon all possible sources of infection.

---

Streissler, of Graz, working at Von Hacker's clinic, has recently published the results of treating old irreducible hip dislocations by the open method, and claims to have had very good results. He accordingly gives the open method the preference in the treatment of such lesions, reserving resection or osteotomy for very severe cases in which it is impossible to secure a functionally good result even by freely laying open the joint and reducing the luxation. The operation has now been done in many cases, but so long as these are not systematically published, good, indifferent, and bad results being frankly brought forward, it is impossible to judge definitely as to its value. Streissler's first case was that of a boy of 14 with an unreduced obturator luxation of five months' duration. The open method, with Kocher's lateral incision, was used, the joint laid open, and the dislocation reduced. The patient made an uninterrupted recovery, and an admirable result was obtained without shortening; movement was free and undisturbed in all directions, and there was no loss of stability. The author ascribes the formation of the new acetabulum, which is so often found in cases of old unreduced luxations, not so much to pressure atrophy as to regenerative activity of the pelvic periosteum. The difficulties are in many cases very great. The head of the bone is atrophied and the relations of the parts are very much disturbed, making the operation in some cases a long and tedious one. Notwithstanding its difficulties, however, Streissler is inclined to lay down the rule that open operation is the method of choice in all cases, and that resection and osteotomy should only be tried as a last resort.

---

The fourth International Thalassotherapeutical Congress will meet at Abbazia from September 28 to 30 next, under the presidency of Professor von Leyden, of Berlin. A very long and interesting programme has been drawn up, which includes the usual clinical meetings and discussions, excursions to neighbouring bathing resorts, and a full inspection of Abbazia itself, which of late years has rapidly risen in importance and now ranks as one of the chief health resorts on the Adriatic. The past three congresses have done good work in drawing the attention of practitioners to the usefulness of seaside resorts, and to the still too-little known possibilities of salt water as a therapeutic agent. Their discussions have stimulated research in various branches of thalasso-therapeutics, and the papers to be read before the Congress this year promise to be as interesting as those which were published during the session of the third Congress. Among the items on the agenda paper we notice, for example, the following papers on questions of general clinical interest: "Indications and Contra-indications for the Sea-bathing Treatment in Cases of Chlorosis and Anæmia" (Dr. Barbier, of Paris, and Dr. Haeberlin, of Wyk), "Sea-bathing in the Treatment of Diseases of Women" (Dr. Lavergne, of Biarritz, and Dr. Sadoveanu, of Costanza), "Dietetics and Hygiene of Thalassotherapy" (Dr. Gmelin, of Foehr), "Analysis of Various Sea Waters with reference to their Therapeutic Value" (Dr. Hennig, of Königsberg), "Climatology in relation to Thalassotherapy" (Professor Glax, of Abbazia). Special tickets at reduced rates will be issued to members of the Congress. For particulars as to membership, etc., practitioners are referred to Professor Julius Glax, Abbazia, the President of the local organisation committee.

